# Competencies of the UK nursing and midwifery workforce to mainstream genomics in the National Health Service: the ongoing gap between perceived importance and confidence in genomics

**DOI:** 10.3389/fgene.2023.1125599

**Published:** 2023-06-16

**Authors:** Catherine Carpenter-Clawson, Melanie Watson, Alison Pope, Kathleen Lynch, Tracie Miles, Dany Bell, Maureen Talbot, Aniko Varadi

**Affiliations:** ^1^ Bristol Royal Hospital for Children, University Hospitals Bristol and Weston NHS Foundation Trust, Bristol, United Kingdom; ^2^South West Genomic Laboratory Hub, North Bristol NHS Trust, Southmead Hospital, Bristol, United Kingdom; ^3^ Genomics Education Programme, Health Education England, Birmingham, United Kingdom; ^4^ NHS Southwest Genomic Medicine Service Alliance, Royal Devon and Exeter Foundation Trust, Exeter, United Kingdom; ^5^ Macmillan Cancer Support, London, United Kingdom; ^6^ British Heart Foundation, Birmingham, United Kingdom; ^7^ Centre for Research in Biosciences, School of Applied Sciences, University of the West of England, Bristol, United Kingdom

**Keywords:** genomics, nurses, midwives, mainstreaming, competencies, National Health Service, workforce, education

## Abstract

The United Kingdom is recognised worldwide as a leader in genomics. The use of genomic technologies in the National Health Service (NHS) is expected to deliver faster and more accurate diagnoses, supporting personalized treatments to improve patient outcomes. The ambition of embedding genomic medicine in the diagnostic pathway requires involvement of the front-line clinical workforce, known as ‘mainstreaming’. Nurses and midwives are the largest professionally qualified workforce in the National Health Service thus, it is anticipated that they will play key roles in mainstreaming. This study investigated the level of competence/confidence of practicing nurses and midwives to support mainstreaming and their perception of the importance of genomics in delivery of patient care. A literature review of genetics/genomics competency frameworks, semi structured interviews of lead nurses and stakeholders were conducted to identify relevant competencies needed for mainstreaming. These were then used to survey four cohorts of nurses (*n* = 153) across England in four consecutive years (2019–22). The confidence level of these professionals in all aspects of genomics was 2.07 ± 0.47 measured on a 5-point Likert scale (1“*Low confidence*”; 5 “*High confidence*”). Intriguingly, these professionals all appreciated the importance of genomics for their patient care (4.01 ± 0.06). Whilst the importance scores increased, the confidence scores declined at the time when major genomic transformation took place in the NHS (e.g.: launch of the Genomic Medicine Service, the National Genomic Test Directory). To bridge this gap, relevant genomic education can play key roles. However, nurses and midwives were found to be grossly underrepresented in formal genomic education courses offered by Health Education England Genomics Education Programme since 2014. This may result from the lack of direct applicability of the currently offered courses for their practice and role. Thematic analysis revealed that nurses and midwives wish to support their patients by providing more information on their condition, inheritance, and treatment options in combination with the use of relevant genetic counselling skills. This study identified easy to follow competencies for embedding genomics into routine clinical care. We propose a training programme that addresses the gap that nurses and midwives currently have, to enable them to harness genomic opportunities for patients and services.

## 1 Introduction

The United Kingdom is a world-leader in genomic medicine starting with Watson and Crick’s discovery of DNA in 1953 and participation in the Human Genome Project (Lander et al., 2001; Venter et al., 2001). In 2012, the United Kingdom Government launched the 100,000 Genomes Project aiming to increase access to whole genome sequencing across the National Health Service (NHS) whilst developing genomic medicine as part of routine healthcare (Smedley et al., 2021). The 100,000 Genomes Project was unique globally in its scale and scope. Recruitment to the project was completed in 2018, data analysis and return of results are now near completion. Access to data for research is part of the on-going legacy of the project (Genomics England, 2022).

Embedding genomic medicine in the mainstream diagnostic pathway to improve patient outcomes in the NHS is now being delivered by the Genomic Medicine Service (GMS) launched at the end of 2018. The GMS aims to offer access to comprehensive genomic testing for all patients in the NHS in England through seven NHS GMS Alliances (GMSAs) geographically aligned to NHS Genetic Laboratory Hubs (GLHs). Genomic tests commissioned by NHS England and the eligibility criteria for patients are all specified now in the National Genomic Test Directory (NGTD) (NHS England, 2022a). The NGTD is updated annually to keep a pace with scientific and technical developments and includes tests for rare and inherited disorders, and cancers.

Routine integration of these genomic tests into clinical care will be carried out by appropriately trained member(s) of the patient’s multidisciplinary clinical team rather than solely by clinical genetics departments (Burton et al., 2010; Al Bakir et al., 2019). Nurses form the single largest group of clinical staff employed in the NHS (National Audit Office, 2021) and their diverse professional roles in a range of different settings make them ideally placed to deliver genomic healthcare (Global Genomics Nursing Alliance, 2022). They also have the potential to lead culture change for implementation of genomics in healthcare, and education of patients and providers about genomic interventions (Williams et al., 2017). Given the potential for the involvement of nurses and midwives in genetic counselling, it is imperative that they are supported by education programs aligned to working practice and increase genomic literacy to deliver patient care. Prior work has been undertaken to identify potential competencies for nurses and midwives in genomic medicine (Jenkins et al., 2001; Calzone, et al., 2002; Kirk et al., 2003; Lewis et al., 2006; Skirton et al., 2010; Kirk, 2013; Boucher et al., 2014; Camak, 2016; Ha et al., 2018), which included aspects of general genetic education, risk assessment processes, clinical referral based on genetic risks, family history taking and an understanding of tumour (somatic) genetic changes, targeted therapies, knowledge on informed consent, ethical, legal, and social implications related to bioethical issues of testing, privacy and security concerns. However, these genetic/genomic competencies were developed prior to the existence of the GMS, when delivery of genomic medicine was principally provided by clinical genetics department.

Health Education England (HEE; which merged with NHS England on the 1st of April 2023) works with partners to plan, recruit, educate, and train the NHS workforce (Health Education England, 2022). To mainstream the findings from the 100,000 Genomes Project into clinical practice HEE launched its Genomics Education Programme (GEP) in 2014 (Genomics Education Programme, 2022). The GEP has developed significantly over the last 8 years with the aim to prepare the multi-disciplinary workforce with the knowledge and skillset to deliver the NHS GMS and to provide genomic education opportunities and resources for the specialist and non-specialist clinical workforce (Table 1). To achieve these educational goals, the GEP has commissioned 7 United Kingdom Universities to run a master’s level qualification in genomic medicine to provide NHS professionals with a multidisciplinary perspective on genomics and its application in healthcare (Genomics Education Programme, 2022). The GEP has also developed a range of education tools, short online courses, and clinical resources (Table 1). Considering all these initiatives it is necessary to understand how well the nursing and midwifery workforce is prepared to contribute to the genomics transformation required by the NHS GMS. This current study investigated the genomic competencies necessary for nurses and midwives to support mainstreaming genomics and their perception of the importance of genomics in the delivery of patient care within the NHS. Based on our findings we are proposing a model of education delivery specifically designed for these professionals.

**TABLE 1:** The GEP resources and tools to facilitate the delivery of genomics education and training.

NHS England Genomic Education Resources/tools	Just-in-time or self-motivation-based resources
1. GeNotes (https://www.genomicseducation.hee.nhs.uk/genotes/)	Yes
2. Bespoke master’s programme: Genomic Medicine Master’s Programme	No
3. Massive Open Online Courses (MOOCs)	No
4. GEP web site educational resources (https://www.genomicseducation.hee.nhs.uk)	Yes
• bite size genomics	
• online courses	
• clinical resources	
• core concepts	
• teaching resources	
• videos	
• podcasts	

## 2 Materials and methods

### 2.1 Data collection

A review of the literature published up to Nov. 2017 was carried out to identify key genomics competencies relevant primarily for cancer Clinical Nurse Specialists (CNSs) and for the nursing workforce in general. The following key words were used for PubMed search: “*nursing competencies” & “genetics and genomics in the UK*” or “*nursing competencies” & “genetics and genomics*”. The former resulted in 57 and the latter 122 hits. Papers that were duplicated following the two separate searches were removed. 15 papers were excluded due to their title alone; abstract/full text were read to assess relevance. Editorials and non-full articles were excluded. Nine papers were used for the development of key competencies in this study (Table 2). The NHS England’s Cancer Strategy (NHS England, 2022b) outlined that all individuals diagnosed with cancer should have a named key worker through their cancer journey and, in most cases, this should be a cancer CNS. It is therefore anticipated that cancer CNSs will need to be confident in discussing genomics and have a clear understanding of its impact on the management of their patients. Thus, the initial investigation regarding genomic competencies focused to this well-defined cohort of nurses in the NHS. Based on the competency list generated from the literature review qualitative semi-structured interviews were then conducted with three senior Lead Cancer Nurses in the West of England Genomic Medicine Centre, which was responsible for delivering the 100,000 Genomes Project across the West of England. The Lead Cancer Nurses rated these competencies 1–10 (1 being “*not relevant*” and 10 being “*highly relevant*”). Key stakeholders including DB (Strategic Advisor Treatment, Medicines and Genomics in cancer care, Macmillan Cancer Support), MT (Head of Clinical Support, Senior Cardiac Nurse, British Heart Foundation), AP (Clinical Lead for Genetic Counselling, Genomics England), AP (Programme Manager, HEE GEP), TM (Associate Director of Nursing and Midwifery, NHS South West Genomic Medicine Service Alliance), MW (Education and Training Lead, South West Laboratory Hub, Fellow of the Higher Education Academy), AV (Senior Fellow of the Higher Education Academy, University of the West of England) then further assessed these competencies for their relevance for all nurse and midwife practitioners within the NHS and across organisations such as the British Heart Foundation and Macmillan Cancer Support. Key skills/competencies that scored above 8 by the Lead Cancer Nurses and approved by stakeholders (Table 2) were included for subsequent quantitative analysis. Quantitative survey was used in four distinct cohorts of nurses: (1) cancer CNSs within the West of England Genomic Medicine Centre, surveyed in July 2019; (2) Continuous Professional Development (CPD) Cohort 1, who signed up to complete the Level 7 (Master level) 15 credit HEE funded module entitled “*Genomic and counselling skills for nurses and healthcare professionals*” in the academic year 2020/21, surveyed in Oct. 2020; (3) CPD Cohort 2 signed up for the same module in the academic year 2021/22, surveyed in Sept. 2021; and (4) CPD Cohort 3 signed up for the same module in the academic year 2022/23, surveyed in Sept. 2022 (Table 3).

**TABLE 2:** Genomic competencies for nurses and midwives essential for GMS delivery in the NHS. These key competencies were identified based on the existing literature (Jenkins et al., 2001; Calzone et al., 2002; Kirk et al., 2003; Lewis et al., 2006; Skirton et al., 2010; Kirk, 2013; Boucher et al., 2014; Camak, 2016; Ha et al., 2018), from qualitative interviews conducted with three Lead Cancer Nurses in the West of England Genomic Medicine Centre delivering the 100,000 Genomes Project for the South West, and with the involvement of key stakeholders (HEE, Macmillan Cancer Support, British Heart Foundation, Genomics England, and Higher Education). The wording of the competencies was intentionally very simple enabling higher education providers to adopt it at various levels (particularly at levels 6 and 7) meeting the Higher Education Qualification descriptors (QAA, 2022).

Competencies
**1.** Understanding of the basic scientific concepts of inheritance, genetics, and genomics
**2.** Understanding of the difference between the germline and somatic genome and clinical implications associated with germline or somatic genetic variants
**3.** Understand what local genetic testing services are available and how to refer patients
**4.** Ability to carry out appropriate risk assessments to identify patients that might be at a higher risk of inherited conditions
**5.** Understand the wider roles and services offered by local clinical genetics teams
**6.** Conduct a comprehensive family history exercise to understand potential high-risk patients for inherited conditions
**7.** Understand the national genetic test directory and its potential relevance for your patients and practice
**8.** Understand the targeted therapies available for patients
**9.** Understand the broad mechanism of action of targeted therapies
**10.** Understand how genomic data can be used in the context of prevention and earlier diagnosis
**11.** Understand how genomic data can be used in the context of patient prognosis
**12.** Understanding how genomic data is analysed and the potential implications of the analysis process on the outcome on patient management
**13.** Understand the wider legal, social, and ethical considerations of genetic testing for patients

**TABLE 3:** Cohorts completed the quantitative and qualitative survey. Cancer CNS nurses completed the survey in July 2019. CPD, Continuous Professional Development course. CPD Cohort 1, Cohort 2 and Cohort 3 signed up to complete the Level 7 (Master level) 15 credit Health Education England funded module entitled “*Genomic and counselling skills for nurses and healthcare professionals*” in academic years 2020/21, 2021/22 and 2022/23.

	Cancer CNS nurses (*n* = 32) July 2019	CPD Cohort 1 (*n* = 30) Oct. 2020	CPD Cohort 2 (*n* = 41) Sept. 2021	CPD Cohort 3 (*n* = 51) Sept. 2022
Specialist Nurse in Cancer Services	32	8	9	25
Specialist Nurse in Cardiac Services	—	6	7	6
Specialist Nurse in Immunology Services	—	1	—	—
Diabetes Specialist Nurse	—	1	1	—
FH Clinical Nurse Specialist	—	—	—	1
Haematology Nurse	—	4	2	4
Mitochondrial disease Specialist Nurse	—	1	—	—
Urology/Renal Clinical Nurse Specialist	—	1	1	1
Specialist Nurse in Neurological Services	—	3	2	—
Midwives	—	2	2	—
Paediatric Genomic Nurse Specialist	—	—	—	3
Research Nurse	—	2	6	1
Staff Nurse	—	—	5	—
Associate Nurse	—	—	—	1
Macmillan Cancer Navigator/Information Specialist	—	1	1	3
Macmillan Professional Development Lead	—	—	—	1
Senior/Lead Nurse involved in GMSAs	—	—	7	5
Regional distribution	Southwest of England	All parts of England and three from Scotland	All seven GMSAs in England	All seven GMSAs in England
Education or training received in genetics or genomics	30%	45%	49%	52%

The cancer CNS cohort was surveyed using a commercial tool “Survey Monkey” Copyright ^©^ 1999–2022 Momentive (SurveyMonkey, 2022) and the questionnaire was replicated for subsequent cohorts using the Joint Information Systems Committee (Jisc) online surveys tool (Online surveys, 2022). All participants from the CPD cohorts filled in the questionnaire before commencing their genomics-related post-graduate Level 7 training (Supplementary Material Survey). For the quantitative survey a Likert scale of 1–5 was used where 1 equated to “*Low confidence*” and 5 equated to “*High confidence*” with a topic. A 5-point Likert scale was also used to gauge the perceived level of importance of the same topics where 1 was equated to “*Not important*” and 5 equated to “*Very important*”; importance was stated to be specifically related to the individual’s current role. All questionnaires provided the option for free-text responses throughout to clarify responses.

Ethical review and approval were not required for the study on human participants in accordance with the local legislation and institutional requirements. The participants provided their written informed consent to participate in this study.

### 2.2 Data analysis

Data was collected from the online survey tools, anonymized and analysed using Microsoft Excel. Student’s t-test was used to compare means between two groups with *p* values equal to the alpha divided by the number of tests (Bonferroni corrected alpha). 95% of confidence interval was estimated by using ±2 standard errors of the mean (SEM; Altman and Bland, 2005). ANOVA was used to compare means between two or more independent groups (Mishra et al., 2019). Normality assumption was checked using Shapiro-Wilk Test (*α* = 0.05). Tukey-Kramer test was used to compare the means of each comparison. Equality variance was assessed by Levene’s test. Kruskal–Wallis Test was also used because the sample size for some groups was just above 30, not all data were normally distributed and there were up to 10% outliers. Post-Hoc Mann Whitney *U* test using a Bonferroni corrected alpha of 0.0083 was used for multiple comparisons (data are shown in Supplementary Tables S1–S4). Seven questionnaires returned with some data missing. The algorithm for missing data suggested by Mirzaei et al. (2022) was followed and given that the missing data was <5% the deletion method was applied. Thus, the entire survey response from the seven respondents that contained any missing data was removed from our analysis. Outliers were defined by using box plots (Supplementary Figures S1–S3) and Mann Whitney *U* Test. Outliers were included in the analysis because these all represented legitimate data and no clear sampling or data entry errors could be identified. Outliers represented up to 10% of the data points and there were no justifiable reasons to remove these points. The Mann Whitley *U* test is robust to the presence of outliers. Thematic analysis of the free-text comments was conducted by using a constant comparison approach (Glaser et al., 1968). Individuals in CPD Cohorts 1, 2 & 3 were also asked on their first introductory tutorial and in a course online interactive discussion forum to explain the key reasons for wanting to learn about genomics. These sessions were recorded, transcribed and key thematic areas, which came up in addition to the free-text comments are listed.

## 3 Results

### 3.1 Identifying and developing key genomics competencies for nurses and midwives in the NHS

**FIGURE 1:**
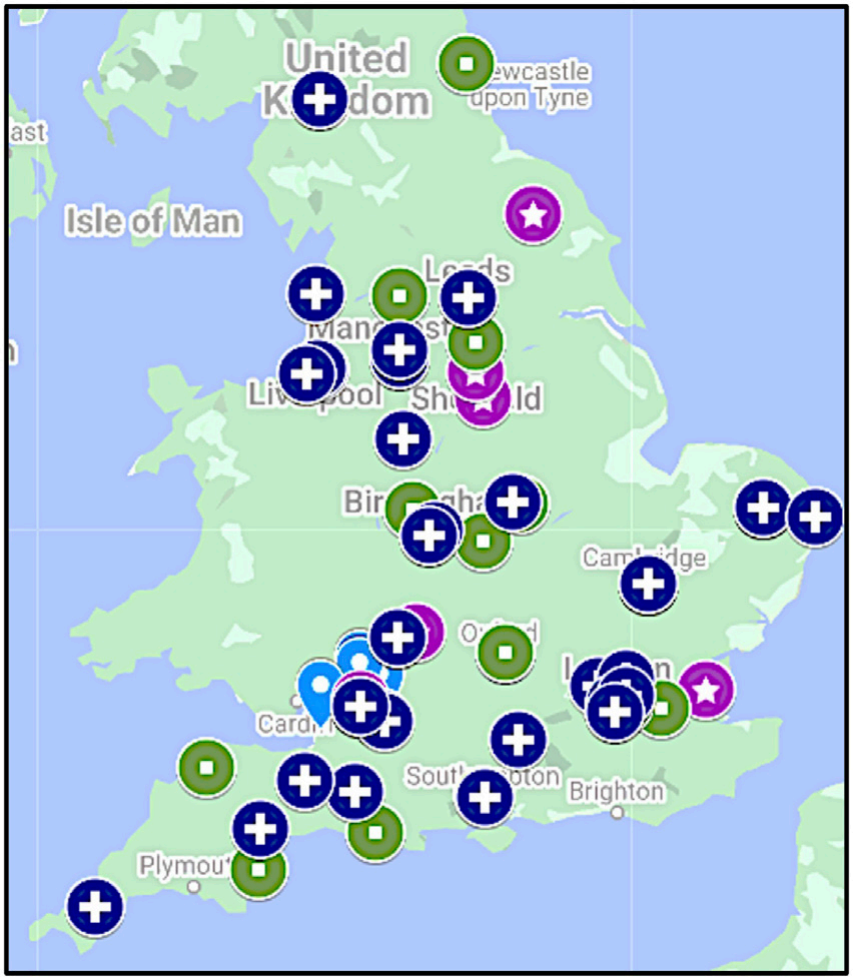
Geographical distribution of cohorts. While cancer CNS nurses were from the South West of England (light-blue drop shape; *n* = 32), CPD Cohort 1 (star in purple circle; *n* = 30), CPD Cohort 2 (square in green circle; *n* = 41) and CPD Cohort 3 (cross in dark blue circle; *n* = 51) were recruited from all parts of England aligned to the seven Genomic Medicine Service Alliances. Note, three participants in CPD Cohort 1 were from Scotland (not shown).

Based on the literature review (Jenkins et al., 2001; Calzone et al., 2002; Kirk et al., 2003; Lewis et al., 2006; Skirton et al., 2010; Kirk, 2013; Boucher et al., 2014; Camak, 2016; Ha et al., 2018) a core very simply worded competency list was generated (Table 2). During the qualitative semi-structured interviews all Lead Cancer Nurses highlighted that “*all are relevant*” and this is a “*comprehensiv*e” list of key genomics competencies (Table 2). The overarching message from the Lead Cancer Nurses was that the topics listed were important, relevant, and covered areas where cancer CNSs would need education and training in future. The same competencies were also assessed by key stakeholders nationally (*n* = 8) for their applicability for all nurse and midwife practitioners within the NHS and across organizations such as the British Heart Foundation and Macmillan Cancer Support. These stakeholders agreed that the list is indeed comprehensive and competencies of healthcare professionals supporting rare and inherited disorders, and cancer, which currently served by the NHS GMS, are all addressed (Table 2). The questionnaire was used to survey four cohorts of nurses and midwives (Table 3). While the cohort of cancer CNS nurses was more homogeneous both in terms of specialism and geographical location, the three CPD cohorts included a broad range of specialisms and represented all regions of England mapping to the seven GMSAs (Table 3; Figure 1). CPD cohorts 2 and 3 had seven and five lead nurses, respectively, who will be or are already involved in the delivery of genomics in their respective NHS Trusts/GMS Alliances.

### 3.2 Confidence of the cohorts in key genomic competencies

**FIGURE 2:**
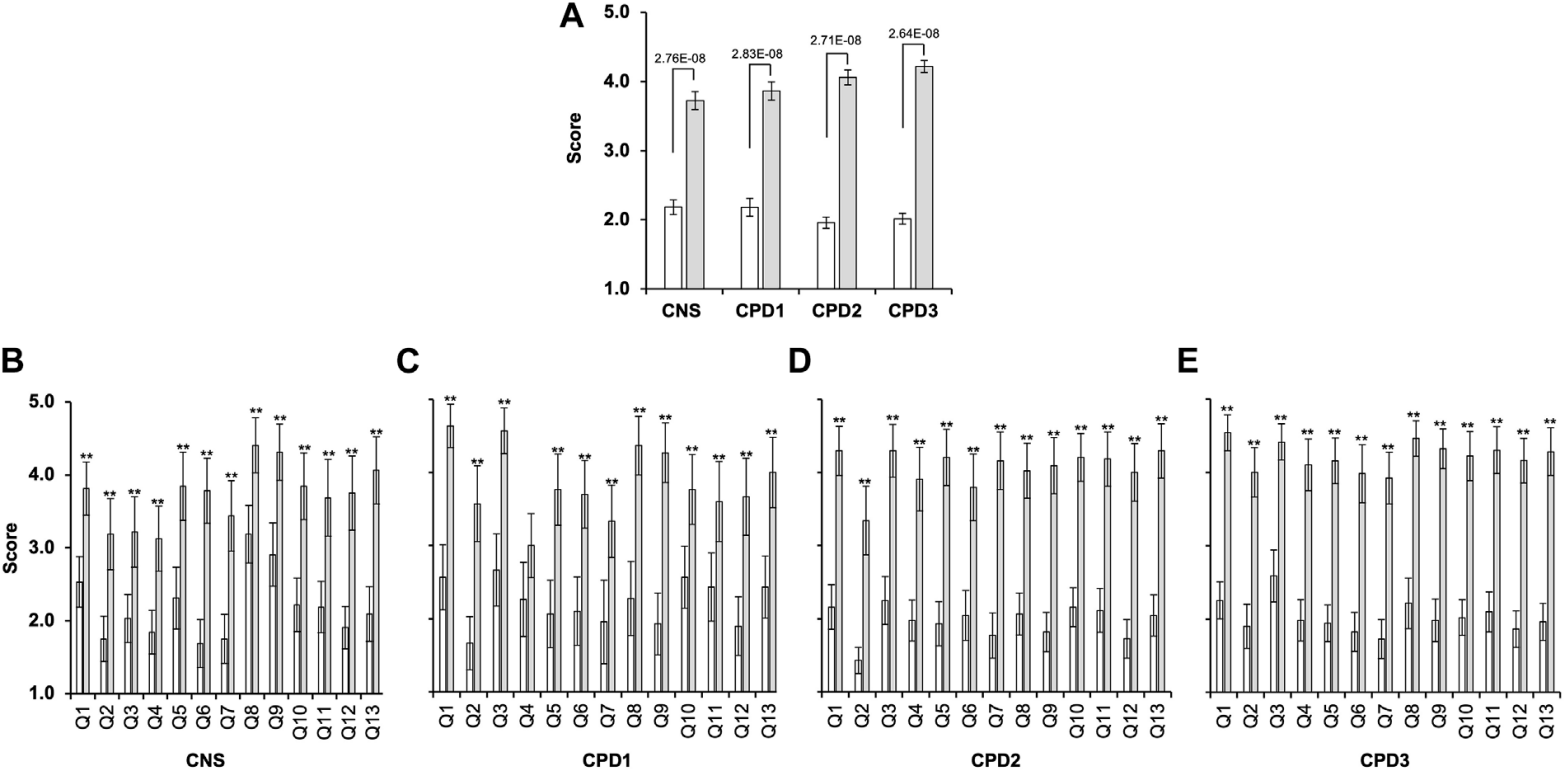
Significant gap exists between confidence and perceived importance of genomics in the nursing and midwifery workforce. Four cohorts of nurses and midwives completed a survey between 2019 and 2022 (Table 2) rating confidence and importance of 13 genomics competencies (Q1-Q13) measured on a 5-point Likert scale where 1 indicated “*Low confidence*” or “*Low importance*” and 5 “*High confidence*” or “*High importance*.” **(A)** Average confidence (white bars) and importance (grey bars) scores for all questions combined with 2 Standard Error of the Mean (2SEM; cancer CNS nurses (*n* = 32); CPD Cohort 1 (CPD1, *n* = 30); CPD Cohort 2 (CPD2, *n* = 41); and CPD Cohort 3 (CPD3, *n* = 51)) (analysed by ANOVA and Tukey Kramer). **(B–E)** Average confidence and importance scores for each question within each cohort. Bonferroni corrected *α* of 0.003846 was used **p < 0.0038 (*t*-test).

**FIGURE 3:**
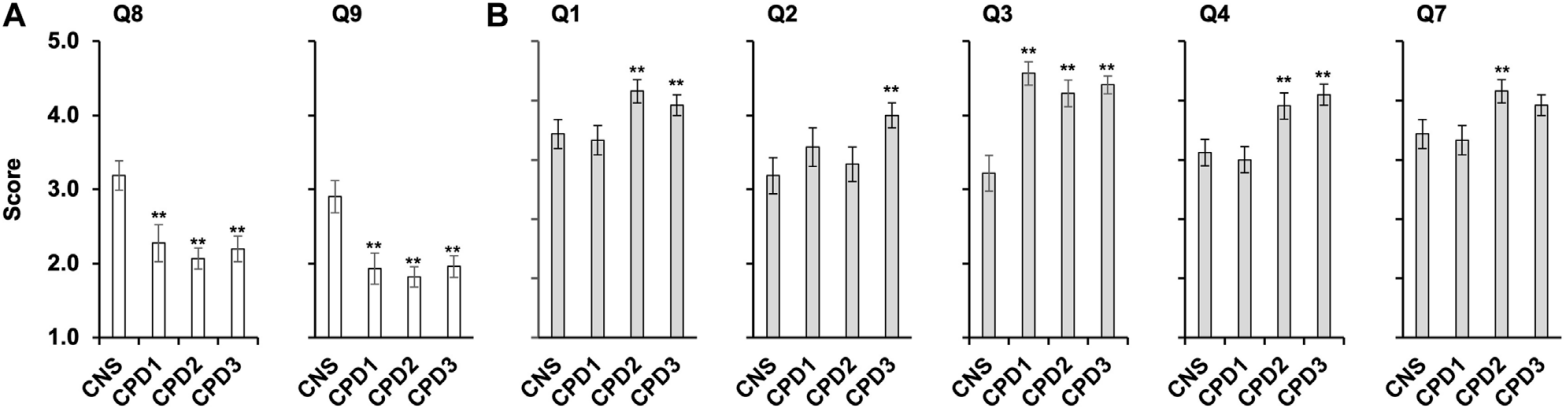
Confidence and importance scores that differ between the cohorts. Four cohorts of nurses and midwives completed a survey (Table 2) rating confidence and importance of 13 genomics competencies (Supplementary Figures S1–S3). **(A)** Average confidence scores for questions 8 and 9 across the four cohorts with SEM, cancer CNS nurses (*n* = 32), CPD cohort 1 (*n* = 30), CPD Cohort 2 (*n* = 41), and CPD Cohort 3 (*n* = 51). Data was analysed using ANOVA and Tukey HSD/Tukey Kramer. ** indicate for Q8 significant difference between the following pairs CNS/CPD1 (*p* = 0.014), CNS/CPD2 (*p* = 0.00031) and CNS/CPD3 (*p* = 0.0014). ** indicate for Q9 significant difference between the following pairs CNS/CPD1 (*p* = 0.0025), CNS/CPD2 (0.00011) and CNS/CPD3 (*p* = 0.00068). No significant difference was observed between CPD1/CPD2, CPD1/CPD3 or CPD2/CPD3 for either Q8 or Q9. **(B)** Average importance scores for questions one to four and 7 with SEM. ** indicate for Q1 significant difference between the following pairs CNS/CPD1 (*p* = 0.0056) and CNS/CPD3 (*p* = 0.0059); ** indicate for Q2 significant difference between the following pair CNS/CPD3 (*p* = 0.0497); ** indicate for Q3 significant difference between the following pairs CNS/CPD1 (*p* = 0.000014); CNS/CPD2 (0.00018) and CNS/CPD3 (*p* = 0.000014). ** indicate for Q4 significant difference between the following pairs CNS/CPD3 (*p* = 0.01); CPD1/CPD2 (0.0195) and CPD1/CPD3 (*p* = 0.0033). ** indicate for Q7 significant difference between the following pairs CPD1/CPD2 (0.042). All other pairs were compared but no significant difference was observed.

The confidence scores in all cohorts were overall very low (Figures 2, 3A). Apart from the cancer CNS cohort’s response to Q8 and Q9 (Figure 3A), for all other questions at least 60% of the respondents scored 1 or 2. Conversely, less than 25% of respondents rated their confidence as either 5 or 4. The average confidence scores were significantly different between the cohorts (*p* = 0.00085, ANOVA). Cancer CNS nurses (2.18 ± 0.10) scores differed compared to CPD2 (1.96 ± 0.08; *p* = 0.00007) and CPD3 cohorts (2.02 ± 0.08; *p* = 0.009). However, no difference was observed between other cohorts when pairwise comparisons were conducted. Confidence scores for each cohort are shown in Figure 2A, white bars. Kruskal–Wallis Test and Mann Whitney *U* test confirmed the above findings (Supplementary Tables S1, S3, S4).

Cancer CNS nurses showed significantly higher confidence in understanding the available targeted therapies (Q8) and their mechanism of action (Q9) than nurses and midwives undertaking the Genomics CPD module (Figure 3A; Supplementary Figure S1). The cancer CNS nurses average score for Q8 was significantly higher 3.19 ± 0.4 *versus* 2.28 ± 0.5 (*p* < 0.005; CPD Cohort 1), 2.07 ± 0.3 (*p* < 0.0001; CPD Cohort 2) or 2.22 ± 0.3 (*p* < 0.0005; CPD Cohort 3). The same was observed for Q9, with average scores 2.91 ± 0.4 *versus* 1.93 ± 0.4 (*p* < 0.005; CPD Cohort 1), 1.80 ± 0.3 (*p* < 0.0001; CPD Cohort 2) or 1.98 ± 0.3 (*p* < 0.0005; CPD Cohort 3). Kruskal–Wallis Test and the post-hoc Mann-Whitney *U* Test also indicated that the mean ranks for Q8 and Q9 were not equal in the four cohorts (Supplementary Table S1)The average confidence scores for Q2 (germline *versus* somatic mutations) was the lowest, only 6 participants scored 4 or 5 out of 153 participants (4%; Supplementary Figure S1B).

85% of all cohorts had no or very little confidence to understand the NGTD and its potential for their practice (Q7). This did not change during the 4-year period when the surveys were conducted (1.75 ± 0.34 for cancer CNS nurses *versus* 1.68 ± 0.25 for CPD Cohort 3, Supplementary Figure S1G).

Individuals in all CPD cohorts were invited to include any comments reflecting their confidence in addition to their numerical scores. Thematic analysis of these comments is shown in Tables 4, 5, 6, 7. This way the Tables would come after Figures 2, 3A as was intended. These reflect the general feeling of these professionals related to genomics, they have no or very little subject knowledge or understanding of the available service within the NHS, which make them uncomfortable and nervous.

**TABLE 4:** Written comments related to confidence in genomics obtained from CPD Cohorts 1–3. Participants were invited to provide comments on their confidence. These comments were grouped in thematic areas and specific examples are listed.

Thematic area	Supporting quote/quotes
I am new **(n = 9)**	“*New to the field of Genomics, my confidence is low across the board*.”
Limited knowledge **(n = 13)**	*“My knowledge around genomics is limited at present. My main objective for this course is to develop my own knowledge to further improve the care I offer to parents, especially in relation to genetic counselling.”*
	*“Genetics is an area I have not had much exposure to since becoming a cardiac nurse. At this point in time my knowledge base is very limited, and I look forward to improving this during the programme.”*
What services exist? **(n = 4)**	*“Very little knowledge on Genomics and services in my area for my patients.”*
I am worried **(n = 8)**	*“Lack of knowledge leaves me feeling nervous and uncomfortable in a rapidly changing field.”*
	“*I am somewhat out of my depth due to lack of knowledge in this area*”
I have the basics **(n = 11)**	*“I feel I have a good basic understanding from a cancer perspective from my current role.”*
I had training recently **(n = 5)**	*“Very recent learning and involvement within this topic.”*

**TABLE 5:** Written comments related to importance of genomics in the CPD Cohorts 1–3. Participants were invited to provide comments on importance of genomics. These were grouped in thematic areas and specific examples are listed.

Thematic area	Supporting quote/quotes
Overarching understanding is important **(n = 25)**	*“I would like to have a more comprehensive and holistic picture of genomics and the services around this field.”*
I want to develop a specific skill **(n = 8)**	“*Carrying out specific genomic-focused tasks, like conducting a comprehensive family history exercise to understand potential high-risk patients for inherited conditions currently is not part of my job role. However, this is an area of development within the team”*
I want to develop my service **(n = 24)**	*“I feel I have very little knowledge within this subject, but greater knowledge and understanding would hugely help improve the service as a whole and help better support patients while ensuring they are better informed.”*
I need to know the services for my patients **(n = 15)**	*“I want to have a good hold on these topics so that I can simplify and demystify them for colleagues and importantly patients so both can have an informed voice.”*

**TABLE 6:** Importance of learning about genomics. CPD Cohorts 1&2 were asked on their first introductory tutorial to explain the key reasons for wanting to learn about genomics. These sessions were recorded, transcribed and key thematic areas, which came up in addition to those in Tables 4, 5 are listed.

Thematic area	Supporting quote/quotes
Learn more about genetic counselling **(n = 28)**	“*I’m hoping to gain some skills in genetic counselling*”
	*“I’m really interested in the counselling side of it, because quite often we are the first professionals who even mention genomics. We have a great team with some counsellors in it, but parents see us first, so it’s really good for us to use correct language and know exactly how to counsel them as a first step.”*
Extend my role and support my patients/parents better **(n = 16)**	“*We often get young people with rare conditions who ask about passing on their disease to any future children. At the moment, I have to pass them on to a doctor to answer this question. So, it’d be really nice to understand their conditions, how they inherit it and what the implications are, just be able to extend my role.*”
	“*This module will help me to support parents because they get very distressed, and they want more information.”*
	*“It will be very helpful for me to support parents”*
	“*I’m usually the first person who parents meet so it will be good to be able to provide more information rather than waiting for a referral.”*
	*“I’m excited to do this because it will give me a greater and more in-depth knowledge to be able to answer patients’ questions, especially around the point of diagnosis.”*
	“*Conversations need to be improved because it’s a fairly new thing for us to be discussing genomics because we normally would just refer* [patients] *on to our local genetic department.*”

**TABLE 7:** Mode of learning about genomics. CPD Cohort 3 was asked on their introductory Discussion Forum to explain the key reasons for signing up for an online genomics course. Note, CPD Cohorts 1&2 started their courses during COVID-19 when they had no choice over the mode of delivery.

Thematic area	Supporting quote/quotes
Online learning provides flexibility and enables participants to study **(n = 35)**	*“It gives a bit more flexibility for learning around a busy full-time job”*
	*“An online course is great, as* [I am] *able to fit it around work and family life.”*
	*“We often have to travel great distances to attend courses, which is difficult to schedule around clinical commitments.”*
I am worried about the technology **(n = 10)**	*“My anxiety though is that I have reached an age where I am beginning to find it more difficult to keep up with technology.”*
	*“I am also starting to struggle with using the technology!”*
	*“My only concern is I am not very tech savvy which is a skill I am also hoping to improve.”*
Access to multi-professionals is easy through the course and support is available **(n = 15)**	“*The online course is great as it involves professionals from all areas of the country, which is great for networking, and it means that I am able to complete the units set at a time convenient for work/life balance.”*
	*“The online course has made access to specialists easier.”*
	*“Interactive discussion forums can be a very useful support.”*
	*“Looking forward to having some live interactive online sessions as this will help share knowledge and give some added focus to the learning.”*

The survey included questions addressing previous formal genetic/genomic education (Table 3). Seventeen of the CNS nurses have been in their role for longer than 5 years and twenty-one (70%) had no formal education or training in genetics and genomics. Similarly, over half of the CPD cohorts had no previous education/training in genetics/genomics. Of those who stated they had received training, the most common areas covered were the basic concepts of inheritance, genetics and genomics, and the targeted therapies and their mechanism of actions available for patients in their specialty area. Some respondents attended day courses on genetics, but many felt that “*sometimes these raised more questions than* [provided] *answers*”. Respondents who disclosed their formal education and training to date, stated that they obtained general nursing education covering basic anatomy, physiology, pathophysiology, and pharmacology.

### 3.3 Key genomics competencies are recognized as highly important for delivery of healthcare in the NHS

Participants were then asked to rate the same competencies for their perceived importance to perform their role effectively and to provide the necessary support for their patients in the NHS. There were no significant differences between the scores within the four cohorts apart for Q1-Q4 and Q7 (Figure 3B; Supplementary Figure S2). CPD Cohorts 1 and 3 rated knowledge and understanding of the basic scientific concepts of inheritance the highest (4.63 ± 0.29 and 4.54 ± 0.24, respectively). This was scored significantly lower by the CNS cohort (3.81 ± 0.36; *p* < 0.001). Although all four cohorts scored Q2 amongst the lowest (Figure 3B; Supplementary Figure S2), CPD cohort 3 rated this significantly higher than the CNS cohort (4.00 ± 0.34 versus 3.18 ± 0.49; *p* < 0.005). Cancer CNS nurses scored Q3, which reflected on their understanding of the local genetic testing services available and how to refer their patients, significantly lower than the other three cohorts (3.22 ± 0.48 *versus* 4.57 ± 0.31, 4.3 ± 0.36 or 4.41 ± 0.25; *p* < 0.0005; Figure 3B). Cancer CNS nurses rated the importance of targeted therapies and their mechanism of action highly 4.41 (Q8) and 4.31 (Q9), respectively (Supplementary Figure S2). At the same time, they scored Q2, the difference between germline and somatic genome, which information can help to select treatment options, the lowest (3.18 ± 0.49; Supplementary Figure S2). Kruskal–Wallis Test and the post-hoc Mann-Whitney *U* Test also indicated that the mean ranks for Q1-Q4 and Q7 were not equal in the four cohorts (Supplementary Table S2).

There was a significant difference between the average confidence and importance scores for all cohorts (Figure 2; Supplementary Table S4). The *p*-values obtained were 2.16E-63 for the cancer CNS cohort, 2.14E-59 for the CPD1 cohort, 2.03E-152 for the CPD2 cohort, and 1.3379E-209 for the CPD3 cohort. Furthermore, there was statistical difference between the confidence and importance scores for each question within all four cohorts (Figure 2B–E).

**FIGURE 4:**
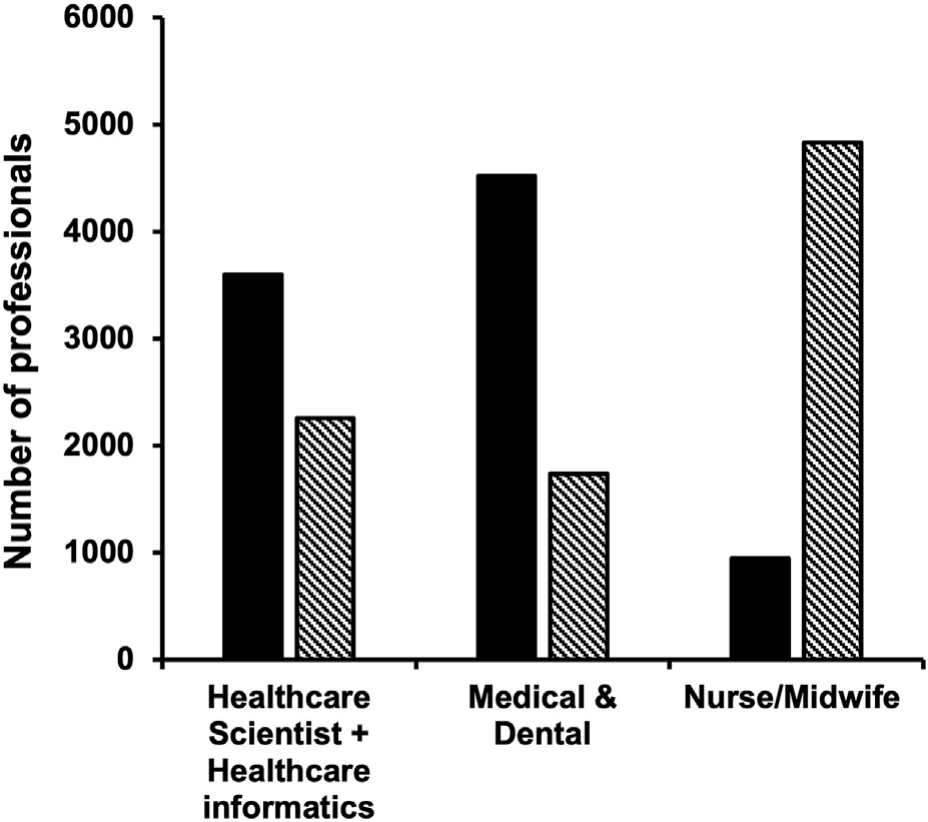
Nurses and midwives are underrepresented in formal genomics training. HEE GEP provided education opportunities to the NHS workforce through master’s level 15-credit modules, which led to various post-graduate qualifications (MSc—180 credits, Post-graduate Diploma—120 credits or Post-Graduate Certificate—60 credits, see also Table 8). Number of professionals who completed 15-credits between 2015 and 31st June 2022 as part of the HEE funded MSc Genomics Medicine Programme is indicated with black bars. Bars with diagonal stripes show numbers that are expected when normalized to the size of each professionally qualified clinical staff in the NHS workforce. Data on was obtained using the NHS Workforce Statistics as of August 2021 (National Audit Office, 2021).

Data from HEE on participation of nurses and midwives in the Genomic Education Master’s in Genomic Medicine Programme up until 31st of June 2022 (Table 8; Figure 4) show underrepresentation of these professionals in funded and credit bearing genomic courses. While 947 15-credit modules were undertaken by nurses and midwives, 3,602 and 4,522 15-credit modules were completed by healthcare scientists and medics, respectively. When numbers are normalised to the workforce size of these professionals in the NHS (Scientific, therapeutic and technical staff 174,773; Nurses and Midwives 373,740; Doctors 134,362 as of Aug. 2021 (National Audit Office, 2021) the underrepresentation and undertraining of nurses and midwives in genomics are very clear (Figure 4).

**TABLE 8:** Professionals completed all or part of the HEE MSc in Genomic Medicine Programme. The number of professionals completed the programme with various credit options over an 8-year period up until 31st of June 2022 are listed. The smallest credit unit within the programme was 15 and this was used to make comparison between the various professional groups (right column). These were then normalised to the workforce size of these professional groups within the NHS (Scientific, therapeutic and technical staff 174,773; Nurses and Midwives 373,740; Doctors 134,362 as of Aug. 2021 (National Audit Office, 2021) shown on Figure 4.

	MSc (180 credit)	PG Dip (120 credit)	PG Cert (60 credit)	CPD (15 credit)	Total (per 15 credit)
Administrative and Clerical	19	3	9	30	**318**
Healthcare Scientist & Health Informatics	235	25	92	214	**3,602**
Medical & Dental	269	37	197	210	**4,522**
Allied Health Professional (AHP)	3	2	1	4	**60**
Nurse & Midwives	54	8	33	103	**947**
Pharmacy	13	7	8	30	**274**
Research	43	4	29	45	**709**
Unknown	19	2	13	12	**308**
Total	648	60	325	518	**10,740**

Total number of professionals who completed 15-credits.

## 4 Discussion

### 4.1 Competencies required for mainstreaming of genomics

This study identified key competencies that are important for nurses and midwives to successfully mainstream genomics into patient care in the NHS and beyond. The study also highlighted the very low overall fundamental knowledge and confidence of these professionals in genomics, but at the same time recognition of its importance for their patient’s care.

The survey was carried out between July 2019—September 2022, when genomics went through a major transformation in the United Kingdom. The 100,000 Genomes Project recruitment had just been completed (Smedley et al., 2021; Genomics England, 2022), the new GMS was created and the NGTD commissioned (Genomics England, 2022). Given that the NHS had hosted the 100,000 Genomes Project since 2012/13 it was anticipated that all healthcare professionals would be very familiar with key genomic concepts by the time the survey was conducted. Despite the progress outlined above, nurses and midwives reported that they did not have the necessary knowledge, understanding and hence, confidence to deliver genomic medicine as part of their day-to-day job in the NHS (Figure 2; Figure 3A; Supplementary Figure S1). Furthermore, the overall awareness of the GMS and the NGTD is remarkably low even in 2022 (Supplementary Figure S1), which highlights the need for wider publicity for this service amongst NHS staff, patients, and the public.

Targeted therapies are very important for the treatment of various cancers (Vanneman and Dranoff, 2012) and cancer CNS nurses demonstrated significantly better understanding of these topics, than the other three cohorts (Figure 3A, Q8 and Q9). However, targeted therapies are also relevant for the management of rare and inherited conditions for example familiar hypercholesterolemia (Hanson, Welch, Frese and Gallo, 2022) or monogenic diabetes (Hattersley and Patel, 2017). Thus, even though the three CPD cohorts were more heterogenous by specialties (Table 3), their confidence in understanding targeted therapies was overall very low. Intriguingly, all cohorts demonstrated very low confidence in “*understanding of the difference between the germline and somatic genome and clinical implications associated with germline or somatic genetic variants*” (Supplementary Figure S1). This is rather surprising particularly for the cancer CNS cohort as this knowledge is very important for cancer patients, their treatments and impact for their relatives (Tsaousis et al., 2021). This suggests that although cancer CNS nurses have some understanding of the use of targeted therapies in their day-to-day practice, but they do not have a clear comprehension of the basis of hereditary cancer treatments for example BRCA1/2-related cancers or hereditary non-polyposis colorectal cancer (HNPCC, also known as Lynch syndrome) (Iyevleva and Imyanitov, 2016). Furthermore, somatic mosaicism is also present in several rare genetic conditions (Tsaousis et al., 2021).

Mainstreaming of genomics involves routine integration of genomic tests into clinical care, which will be carried out by a front-line clinical workforce rather than solely by clinical genetic departments. In this model, non-genetic practitioners are expected to identify at risk individuals, initiate genetic discussions, take a family history, assess the chance of a genetic condition, organise genetic testing, and/or deliver a genetic test result to a patient. There is growing evidence of successful and rapid dissemination of genetic information into routine clinical care in nurse-led services such as diabetes services in the United Kingdom (Shepherd et al., 2014) and a Mainstreaming Cancer Genetics (MCG) service for BRCA1/2 at the Nottingham University Hospitals NHS Trust (Scott et al., 2020). Considering that nurses and midwives are the largest professionally qualified clinical workforce (about 50%) in the NHS (National Audit Office, 2021), it is unlikely that mainstreaming can be successfully done without providing practice-relevant training to this group of professionals. This is supported by the fact that all our cohorts recognized the importance of genomics for their patient’s care (Figure 2; Supplementary Figure S2).

### 4.2 Training of nurses in science, genetics, and genomics

Those who completed the survey left formal education at least 5 years ago, with many completing education some 15–26 years previously. While they received training in basic anatomy, physiology, pathophysiology, pharmacology, only a few had education/qualifications in genetics or genomics. Twelve Lead Nurses involved in GMSA signed up for the second and third CPD cohorts (Table 3), and despite their regional leading roles they had limited knowledge and training in genomics. Close to 11,000 HEE GEP 15-credit master-level modules were funded up to June 2022, but less than 1/10 of these were completed by nurses/midwives (Table 8). If the size of this workforce in the NHS is considered, this number would need to be at least 5-7-fold higher (Figure 4). The Master’s in Genomic Medicine programme contains several core/compulsory modules, which are not relevant for the day-to-day work of nurses and midwives such as Omics Techniques and Technologies, Bioinformatics, Interpretation and Data Assurance (Genomics Education Programme, 2022). Thus, it is not surprising that only 54 nurses and midwives completed the full MSc *versus* 504 healthcare scientists, medics and dentists. These highlight that practicing nurses and midwives need formal training in genetics and genomics, but this needs to be directly relevant and applicable for their practice and role.

### 4.3 Proposed content and delivery model of genomic education of nurses and midwives

**FIGURE 5:**
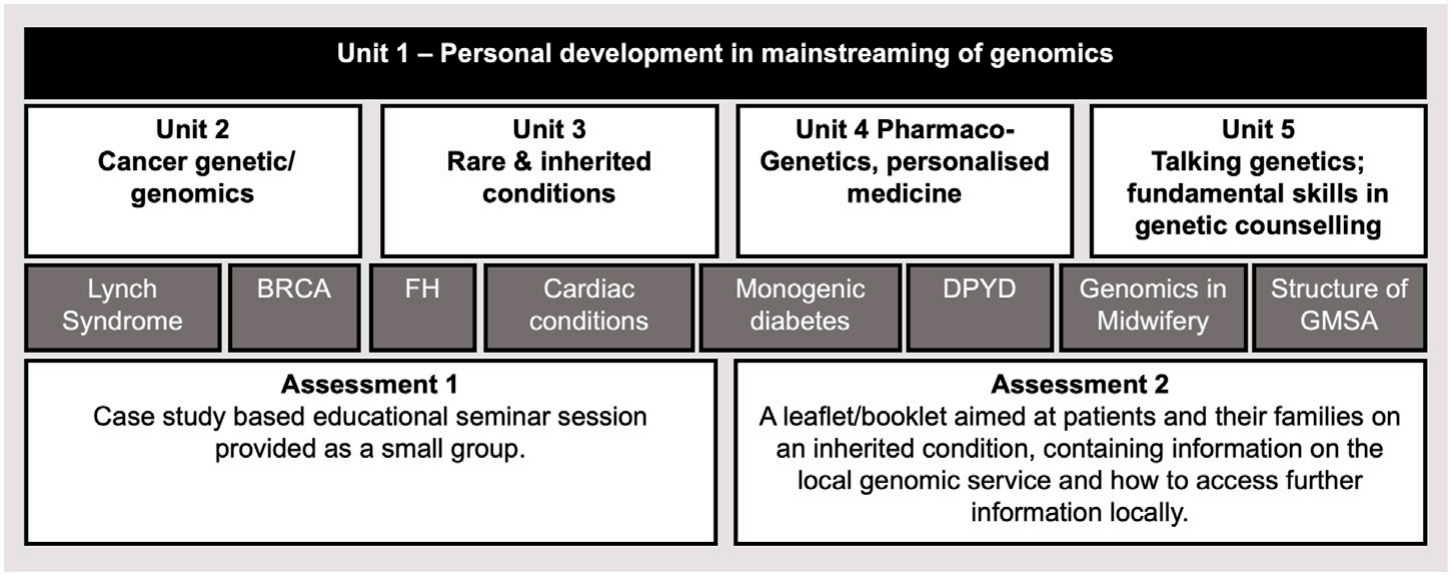
Proposed master’s level 15 credit module structure to train competent nurses and midwives in genomics. Unit 1 prepares nurses and midwives for developing their leadership, communication and train the trainer skills and behaviours, which support effective mainstreaming of genomics. This Unit also introduces the GMS structure and how genomics is delivered at local, regional, and national levels to enhance their overall organisational competence. Units 2-4 develop their genetics and genomics subject knowledge relevant for their profession. Unit 5 enhances their skills to communicate genomic aspects with their peers, patients, and their families. Some topics, which are used as case studies, and currently delivered as part of the GMS National Transformation Projects, are listed in dark grey rectangles, BRCA, Breast Cancer Gene; FH, Familial hypercholesterolaemia; DPYD, Dihydropyrimidine Dehydrogenase; GMSA, Genomic Medicine Service Alliance. Assessments are aimed to test participants critical thinking, application, and implementation of the skills that they gain during the module. We propose that lectures are prerecorded and available online, these sessions are supported with online-live interactive tutorials and online discussion forums.

The in-person survey revealed that effective patient-centered communication and genetic counselling skills, which facilitate patient support and decision making, were seen by our participants as key additional competencies for mainstreaming (Table 6). We thus, devised a postgraduate level 7 module (Figure 5.) that addresses the gap that these professionals currently have (Tables 2, 6). We created a “*Talking Genetics*” unit recognizing that aspects of genetic counselling will become part of the mainstreamed health service, but some other aspects will be expected to remain within the specialist genetics workforce (e.g.: implications for future health and familial consequences; Patch and Middleton, 2018). Understanding the fundamentals of genetics and genomics was scored as one of the most important competencies by all our professionals. Previous studies also reported low fundamental knowledge of nurses in genomics (Calzone et al., 2018; Bueser et al., 2022). Therefore, our course includes three units “*Cancer Genetic/Genomics*”, “*Rare and Inherited conditions*” and “*Pharmacogenetis*”, which enhance their genomic literacy and application of genomics in their clinical practice (Figure 5). In our module the key genetic/genomic concepts are illustrated via practice relevant genetic conditions (Figure 5. Dark grey rectangles) linked to tailored communication and appreciation the rights of all individuals to make their own informed decisions and voluntary action.

The module is a 20–25 weeklong online course that includes synchronous virtual discussions (lectures and participants meet at the same time) and asynchronous virtual interaction within the cohort as well as with lectures. We followed Johnson and Aragon (2003) seven principles to provide an effective online learning environment: ‘(1) address individual differences, (2) motivate the student, (3) avoid information overload, (4) create a real-life context, (5) encourage social interaction, (6) provide hands-on activities, and (7) encourage student reflection’. This module can be integrated into other Master’s level programmes or can be used as a credit bearing Continuous Professional Development (CPD) course.

To date sixty-five professionals completed and fifty-two have just commenced this new module in Autumn 2022. Impact on their competence and confidence in delivering genomics as part of routine care immediately and 12 months after completing the course using the Kirkpatrick four-level training evaluation model (Falletta, 1998) is currently being conducted. Our pilot data obtained from CPD cohorts 1&2 (*n* = 46) indicate behavioral change, application of the newly gained skills to practice and service improvements through the development of, for example new nurse-led BRCA and Lynch services (manuscript in preparation). Further evidence of the effectiveness is the popularity of our course, which was 100% oversubscribed in its third intake; the commissioning and successful validation of a new Postgraduate Certificate in Genomics programme that expands the original module with further training in communication, genetic counselling and setting up nurse-led genomic services with service evaluation. Our framework also informs the HEE genomics competency for the nursing, midwifery, and health visiting professions which is currently open for consultation (NHS choices, 2022).

Simpson et al. (2019) conducted a national survey of workforce education in genomics in the NHS. This revealed a significant preference for online learning, but a large proportion still favored face-to-face delivery. Our CPD cohort 3 had clear preference for online learning due to its easy accessibility and flexibility (Table 7). Considering the large number of nurses and midwives in the NHS who would need upskilling in genomics, a Massive Open Online Course (MOOC) delivery model seems very attractive. MOOCs were anticipated to provide a disruptive transformation in postsecondary education, but the completion rates of these courses have been low with certification rates between 2%–10% (Rai and Chunrao, 2016; Reich and Ruipérez-Valiente, 2019). The main reasons for discontinuation of studies are reported as the lack of live instructor involvement, no student-teacher engagement, and no follow-up or feed-back to students on learning. Research suggests that human connections through tutors and peer groups provide the most important student support (Reich and Ruipérez-Valiente, 2019). A MOOC course ‘*Whole Genome Sequencing: Decoding the Language of Life and Health*' on the FutureLearn platform has been developed by the GEP with the aim to support the professional development of the healthcare workforce at scale (Bishop et al., 2019). Almost 20,000 participants enrolled on the course, 45% viewed at least one step, 29% engaged in the discussion with other participants at least once and only 100 participants, who responded to the post-course survey, were from the NHS. Although MOOC is a good vehicle to reach large number of learners, as suggested by Reich and Ruipérez-Valiente (2019) they are most valuable in providing niche courses to already educated learners. As we showed, the nursing and midwifery workforce almost completely lacks genomic education and thus, online-only short, or longer courses without live and interactive lecturer engagement and problem-solving opportunities are not well suited for this group of professionals. Indeed, although all four Kolb’s learning styles exists among nurses both in traditional classroom settings and online, the *accommodators-divergers* are the predominant styles preferring concrete experiences, interested in people and like working in teams (Smith, 2010). Access to CPD is a priority for the NHS, however professionals are less willing to complete these courses in their own time (Simpson et al., 2019). Thus, it is vitally important that nurses and midwives are supported to have protected study leaves that enables them to engage with genomics delivered by courses that consider their preferred learning styles.

Limitation of our study is that no detailed demographics (e.g.: age, type of formal previous education, graduation time, working experience) were available for all four cohorts that may impact on the confidence and importance scores. Surveys are very useful to gather information from a large cohort, but these normally lack explanative responses. For us to design a useful education programme, responses from tutorial sessions and discussion forums (Tables 6, 7) were very important, which undoubtably limited our sample size. Wide practicing areas of the survey takers (Table 3) might also influence the quantitative scores. However, the professionals’ broad clinical roles are representative of those who are expected to be involved in mainstreaming genomics in a range of healthcare settings.

## 5 Conclusion

Mainstreaming of genomics requires the upskilling of nurses and midwives, the largest professionally qualified clinical workforce of the NHS. These professionals, although recognising the importance of genomics for their patient care, do not currently have the basic subject knowledge, understanding and confidence that would enable them to integrate genomics into their service. Identification of a simple competency framework enables Higher Education Institutions to develop/adopt a postgraduate training course, which can be delivered online supported by tutors and peer-group through synchronous and asynchronous virtual interactions, follow-up and feed-back on learning that fully meet the preferred learning styles of these professionals. It is very important to enable these professionals, who are frequently the first contact for patients and their families, to understand the genetic aspect of their patients’ condition, conduct the relevant consenting for genetic testing, communicate confidently the potential targeted treatments and implications for family members. Effective nurse-led services have the potential to transform healthcare through improving patient experience, reducing waiting times and overall costs. Our ambition to collaborate with the Global Genomics Nursing Alliance (G2NA; www.g2na.org; Calzone et al., 2018) and through working with the NHS England Workforce, Training and Education Directorate (formerly HEE) Global Health Partnerships team will enable dissemination and adaptation of our proven education model globally.

## Data Availability

The raw data supporting the conclusion of this article will be made available by the authors, without undue reservation.

## Supplementary material

SUPPLEMENTARY FIGURE S1 Distribution of confidence scores for each question (Q1-13) among the four cohorts. Within each box, horizontal lines denote median values; x indicates sample average; boxes extend from the 25th to the 75th percentile of each cohort’s distribution values; the box length shows the interquartile range (IQR); the whiskers extend 1.5 times the IQR; dots denote outliers, which are observations outside the maximum and minimum data values. *p*-values were obtained by ANOVA.

SUPPLEMENTARY FIGURE S2 Distribution of importance scores for each question (Q1-13) among the four cohorts. Within each box, horizontal lines denote median values; x indicates sample average; boxes extend from the 25th to the 75th percentile of each cohort’s distribution values; the box length shows the interquartile range (IQR); the whiskers extend 1.5 times the IQR; dots denote outliers, which are observations outside the maximum and minimum data values. *p*-values were obtained by ANOVA.

SUPPLEMENTARY FIGURE S3 Distribution of confidence (white) and importance (grey) scores within each cohort. Within each box, horizontal lines denote median values; x indicates sample average; boxes extend from the 25th to the 75th percentile of each cohort’s distribution values; the box length shows the interquartile range (IQR); the whiskers extend 1.5 times the IQR; dots denote outliers, which are observations outside the maximum and minimum data values. *p*-values were obtained by ANOVA.
