# Supplementary figures and images for: Competencies of the UK nursing and midwifery workforce to mainstream genomics in the National Health Service: the ongoing gap between perceived importance and confidence in genomics
Source: Front Genet. 2023 Jun 16;14:1125599. doi: 10.3389/fgene.2023.1125599 (PMC10312078; doi:10.3389/fgene.2023.1125599)

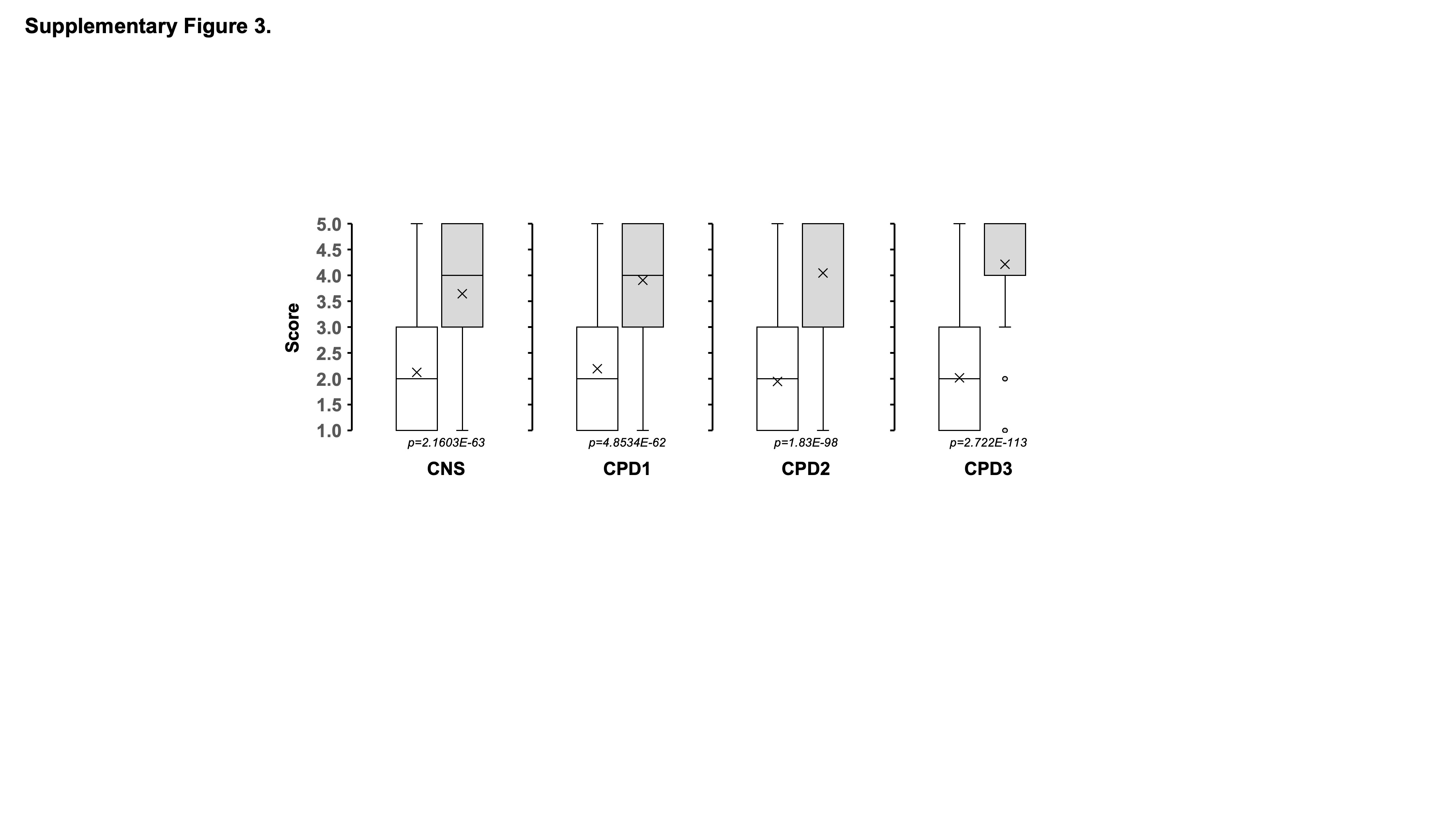

Supplement: Supplementary file 1 [file Image3.JPEG]

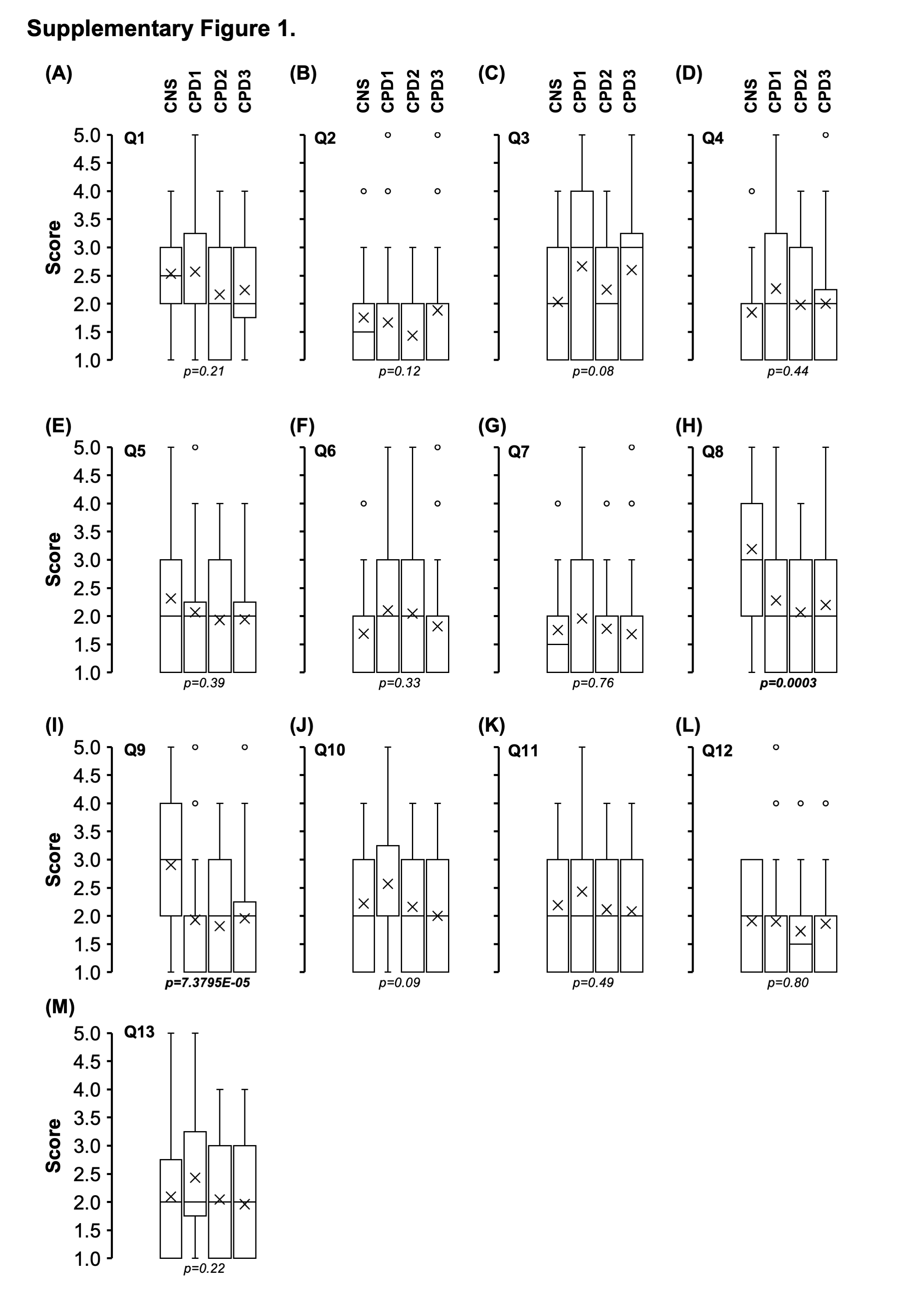

Supplement: Supplementary file 2 [file Image1.TIFF]

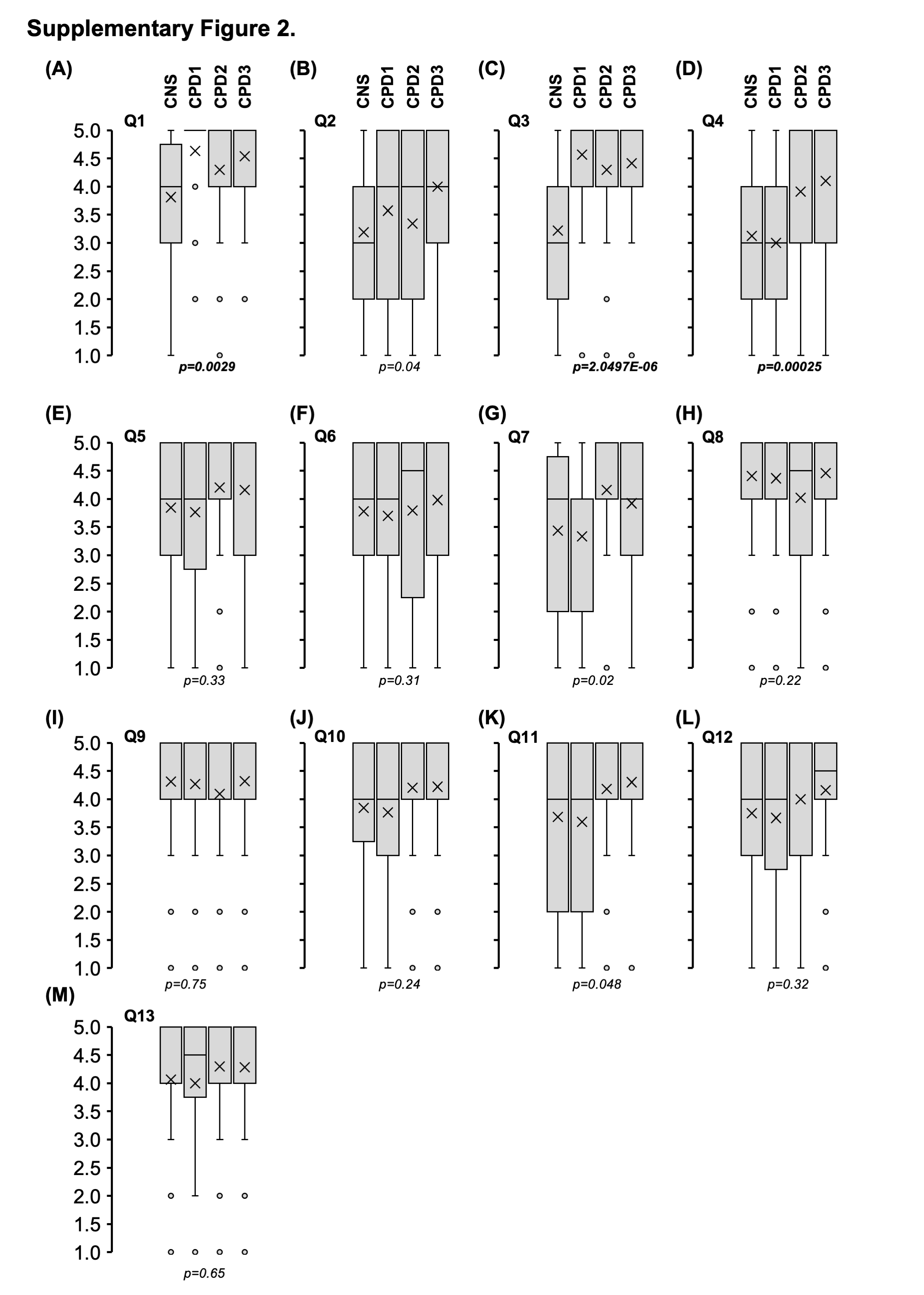

Supplement: Supplementary file 4 [file Image2.TIFF]
