# Supplementary material for: Competencies of the UK nursing and midwifery workforce to mainstream genomics in the National Health Service: the ongoing gap between perceived importance and confidence in genomics
Source: Front Genet. 2023 Jun 16;14:1125599. doi: 10.3389/fgene.2023.1125599 (PMC10312078; doi:10.3389/fgene.2023.1125599)
Supplement: Supplementary file 3 [file Table1.DOCX]

**Supplementary Table 1. Non-parametric analysis of confidence scores.** Box plot analysis (Supplementary Figs 1-3) revealed that there were up to 10% outliers. To carry out robust analysis non-parametric tests were used. Kruskal-Wallis Test, also called one-way ANOVA on ranks, was performed and the p-values in bold indicate that some of the group’s mean ranks were not equal (e.g.: Q8 and Q9). The Post-hoc Mann-Whitney U Test using a Bonferroni corrected alpha of 0.0083 indicated that the mean ranks of some of the pairs were significantly different (numbers in bold). The normality was checked based on the Shapiro-Wilk Test (α=0.05).

|  | Q1 | Q2 | Q3 | Q4 | Q5 | Q6 | Q7 | Q8 | Q9 | Q10 | Q11 | Q12 | Q13 |
| --- | --- | --- | --- | --- | --- | --- | --- | --- | --- | --- | --- | --- | --- |
| Kruskal-Wallis Test p-value | 0.35 | 0.22 | 0.12 | 0.84 | 0.54 | 0.47 | 0.99 | **0.00039** | **0.00038** | 0.22 | 0.74 | 0.71 | 0.37 |
|  | Mann-Whitney U Test p-values (corrected a=a/m=0.05/6= 0.008333) | | | | | | | | | | | | |
| CNS/CPD1 | 0.84 | 0.53 | 0.06 | 0.41 | 0.30 | 0.20 | 0.92 | **0.004434** | **0.001499** | 0.25 | 0.57 | 0.58 | 0.23 |
| CNS/CPD2 | 0.12 | 0.14 | 0.40 | 0.54 | 0.18 | 0.17 | 0.90 | **5.036E-05** | **0.0001225** | 0.95 | 0.76 | 0.27 | 0.97 |
| CNS/CPD3 | 0.21 | 0.72 | 0.05 | 0.56 | 0.22 | 0.42 | 0.80 | **0.0003657** | **0.0005509** | 0.42 | 0.63 | 0.67 | 0.71 |
| CPD1/CPD2 | 0.21 | 0.49 | 0.05 | 0.61 | 0.85 | 0.93 | 0.99 | 0.81 | 0.88 | 0.19 | 0.38 | 0.61 | 0.19 |
| CPD1/CPD3 | 0.40 | 0.31 | 0.80 | 0.70 | 0.93 | 0.48 | 0.92 | 0.92 | 0.76 | 0.04 | 0.29 | 0.82 | 0.08 |
| CPD2/CPD3 | 0.64 | 0.04 | 0.21 | 0.92 | 0.83 | 0.43 | 0.89 | 0.88 | 0.59 | 0.36 | 0.86 | 0.40 | 0.64 |

**Supplementary Table 2. Non-parametric analysis of importance scores.** Box plot analysis (Supplementary Figs 1-3) revealed that there were up to 10% outliers. To carry out robust analysis non-parametric tests were used. Kruskal-Wallis Test, also called one-way ANOVA on ranks, was performed and the p-values in bold indicate that some of the group’s mean ranks were not equal (e.g.: Q1-Q4 and Q7). The Post-hoc Mann-Whitney U Test using a Bonferroni corrected alpha of 0.0083 indicated that the mean ranks of some of the pairs were significantly different (numbers in bold). The normality was checked based on the Shapiro-Wilk Test (α=0.05).

| **Importance** | Q1 | Q2 | Q3 | Q4 | Q5 | Q6 | Q7 | Q8 | Q9 | Q10 | Q11 | Q12 | Q13 |
| --- | --- | --- | --- | --- | --- | --- | --- | --- | --- | --- | --- | --- | --- |
| Kruskal-Wallis Test p-value | **0.00014** | **0.05** | **0.000012** | **0.00005** | 0.25 | 0.51 | **0.0050** | 0.25 | 0.89 | 0.14 | 0.09 | 0.46 | 0.48 |
|  | Mann-Whitney U Test p-values (corrected α=α/m=0.05/6= 0.008333) | | | | | | | | | | | | |
| CNS/CPD1 | **0.00020** | 0.27 | **2.65E-05** | 0.69 | 0.79 | 0.76 | 0.73 | 0.86 | 0.85 | 0.77 | 0.79 | 0.79 | 0.82 |
| CNS/CPD2 | 0.00900 | 0.56 | **0.000210** | **0.00430** | 0.15 | 0.61 | **0.00638** | 0.11 | 0.46 | 0.17 | 0.15 | 0.40 | 0.26 |
| CNS/CPD3 | **0.00010** | **0.007** | **3.03E-05** | **0.00066** | 0.29 | 0.27 | 0.09 | 0.74 | 0.74 | 0.11 | 0.08 | 0.28 | 0.43 |
| CPD1/CPD2 | 0.14 | 0.58 | 0.45 | **0.00127** | 0.09 | 0.43 | **0.00227** | 0.16 | 0.60 | 0.10 | 0.08 | 0.27 | 0.18 |
| CPD1/CPD3 | 0.57 | 0.17 | 0.34 | **0.00015** | 0.18 | 0.17 | 0.04 | 0.90 | 0.92 | 0.06 | 0.04 | 0.17 | 0.30 |
| CPD2/CPD3 | 0.26 | 0.04 | 0.93 | 0.74 | 0.55 | 0.54 | 0.17 | 0.10 | 0.61 | 0.83 | 0.76 | 0.84 | 0.68 |

**Supplementary Table 3. Non-parametric analysis of confidence and importance scores.** Mann-Whitney U Test was used to carry out pairwise comparisons between or within cohorts.

|  | Confidence | | Importance | |  | Confidence/Importance |
| --- | --- | --- | --- | --- | --- | --- |
| Cohorts | Mann Whitney U test  p-value | Tukey Fence | Mann Whitney U test  p-value | Tukey Fence | Cohorts | Mann Whitney U test |
| CNS/CPD1 | 0.2918 | No outliers | 0.9999 | No outliers | CNS | Conf and Imp populations are not equal; statistically significant |
| CNS/CPD2 | **0.002607** | No outliers | **1.399E-05** | No outliers | CPD1 | Conf and Imp populations are not equal; statistically significant |
| CNS/CPD3 | **0.01502** | No outliers | **1.138E-10** | No outliers | CPD2 | Conf and Imp populations are not equal; statistically significant |
| CPD1/CPD2 | 0.07502 | No outliers | **0.002947** | No outliers | CPD3 | Conf and Imp populations are not equal; statistically significant |
| CPD1/CPD3 | 0.2918 | No outliers | **1.102E-05** | 8.3% CPD3 | All cohorts together | Conf and Imp populations are not equal; statistically significant |
| CPD2/CPD3 | 0.4505 | No outliers | 0.2122 | 8.44% CPD3 |  |  |

**Supplementary Table 4. Non-parametric analysis of importance and confidence scores within each cohort.** Mann-Whitney U Test was carried out comparing confidence and importance scores within each cohort. The p values where mean ranks were significantly different are indicated in bold. The distribution was approximately normal. For comparison, ANOVA p-values are indicated in the second rows in each column.

|  | Q1 | Q2 | Q3 | Q4 | Q5 | Q6 | Q7 | Q8 | Q9 | Q10 | Q11 | Q12 | Q13 |
| --- | --- | --- | --- | --- | --- | --- | --- | --- | --- | --- | --- | --- | --- |
| CNS Imp/Conf | **<0.00001**  3.57E-06 | **<0.00001**  5.50E-06 | **0.00068**  1.35E-03 | **0.0001**  1.17E-05 | **<0.00001**  8.82E-06 | **<0.00001**  2.41E-10 | **<0.00001**  3.10E-07 | **<0.00001**  3.71E-05 | **<0.00001**  8.84E-06 | **<0.00001**  6.84E-07 | **0.00012**  1.33E-05 | **<0.00001**  3.32E-08 | **<0.00001**  8.19E-09 |
| CPD1  Imp/Conf | **<0.00001**  1.80E-10 | **<0.00001**  1.11E-07 | **<0.00001**  1.91E-08 | 0.16452  0.18350 | **<0.00001**  4.19E-06 | **0.00008**  1.02E-05 | **0.00108**  0.00046 | **<0.00001**  2.96E-09 | **<0.00001**  5.47E-12 | **0.00084**  0.00066 | **0.00438**  0.00196 | **<0.00001**  7.31E-07 | **<0.00001**  8.40E-06 |
| CPD2  Imp/Conf | **<0.00001**  6.46E-15 | **<0.00001**  3.05E-11 | **<0.00001**  7.13E-13 | **<0.00001**  6.23E-11 | **<0.00001**  9.85E-15 | **<0.00001**  2.34E-08 | **<0.00001**  4.93E-15 | **<0.00001**  1.53E-12 | **<0.00001**  1.98E-15 | **<0.00001**  2.36E-15 | **<0.00001**  2.03E-13 | **<0.00001**  3.41E-15 | **<0.00001**  2.45E-15 |
| CPD3  Imp/Conf | **<0.00001**  2.94E-23 | **<0.00001**  3.32E-15 | **<0.00001**  3.36E-13 | **<0.00001**  3.69E-15 | **<0.00001**  4.3E-19 | **<0.00001**  1.15E-14 | **<0.00001**  1.63E-17 | **<0.00001**  4.83E-18 | **<0.00001**  1.12E-20 | **<0.00001**  2.94E-18 | **<0.00001**  7.22E-18 | **<0.00001**  2.66E-20 | **<0.00001**  1.16E-19 |
