# Supplementary material for: Competencies of the UK nursing and midwifery workforce to mainstream genomics in the National Health Service: the ongoing gap between perceived importance and confidence in genomics
Source: Front Genet. 2023 Jun 16;14:1125599. doi: 10.3389/fgene.2023.1125599 (PMC10312078; doi:10.3389/fgene.2023.1125599)
Supplement: Supplementary file 5 [file DataSheet1.docx]

Genomics CPD - Survey 1 – Pre-course benchmark

Top of Form

Page 1: The Survey

**Evaluation of course**

This is the third time we have provided this course in this format, and we are continually seeking opportunities to improve the experience of students; we would appreciate your considered reflection so that we can make improvements prior to running the course again.  Completing this feedback should also assist in your own reflection and therefore we expect students to complete all elements of the course evaluation questionnaires.

**GDPR and data usage**

The data you provide will be anonymised.

The data will be used for analysis for ideas for course improvements and may be used as evidence in educational publications.  The data will be kept for a maximum of two years and curated on this Jisc online survey tool which complies with GDPR regulations.

- In continuing with survey, we take this as your consent for data usage.
- It should take no more than 10 minutes to complete.

*Thank you in advance* *from the course team*

**Evaluation Part 1 - Pre learning education needs assessment**

This questionnaire will give us a baseline as to your educational need and perception of genomics.  You will complete this twice - at the very beginning of the course and at the end.

It will also give you an indication of the level of learning achieved by participating in the course.  This will help you in your reflection of the course.

This questionnaire will require you to complete your name so that we can match pre- and post- course responses.  The data will then be anonymised.

Please click **next** when you are ready to begin

Page 2: About you

*1.*Please enter your UWE user name.  The one that you will use to log in to 'My UWE'  *For example Mel Watson - my UWE username is mj-watson.  If you don’t have this, please simply enter your name.* *Required*

*2.*Please state your role and main work base. *For example:  Cancer Nurse Specialist, Great Ormond Street Hospital* *Required*

*3.*Please state the clinical speciality that you currently work in. E.g. Cancer - Gynaecology or Cardiac etc. *Required*

Page 3: Using scales for self-reflection on Genomic Topics

**Current level of expertise in Genomic topics**

*4.***Please review each of the topics listed below.**
On a scale of 1 to 5; where 1*is 'low confidence​'*and *5 is 'high confidence​'*; how confident are you regarding your current level of expertise in this topic? *Required*

Please don't select more than 1 answer(s) per row.

Please select at least 13 answer(s).

|  | 1 (low confidence) | 2 | 3 | 4 | 5 (high confidence) | Not applicable |
| --- | --- | --- | --- | --- | --- | --- |
| Understanding of the basic scientific concepts of inheritance, genetics and genomics |  |  |  |  |  |  |
| Understanding of the difference between the germline and somatic (tumour) genome and clinical implications associated with germline or somatic genetic variants |  |  |  |  |  |  |
| Understand what local genetic testing services are available and how to refer patients |  |  |  |  |  |  |
| Ability to carry out appropriate risk assessments to identify patients that might be at a higher risk of inherited conditions |  |  |  |  |  |  |
| Understand the wider roles and services offered by local clinical genetics teams |  |  |  |  |  |  |
| Conduct a comprehensive family history exercise to understand potential high-risk patients for inherited conditions |  |  |  |  |  |  |
| Understand the national genetic test directory and its potential relevance for your patients and practice |  |  |  |  |  |  |
| Understand the targeted therapies available for patients |  |  |  |  |  |  |
| Understand the broad mechanism of action of targeted therapies |  |  |  |  |  |  |
| Understand how genomic data can be used in the context of prevention and earlier diagnosis |  |  |  |  |  |  |
| Understand how genomic data can be used in the context of patient prognosis |  |  |  |  |  |  |
| Understanding how genomic data is analysed and the potential implications of the analysis process on the outcome on patient management |  |  |  |  |  |  |
| Understand the wider legal, social, and ethical considerations of genetic testing for patients |  |  |  |  |  |  |

*a.* Please add any comments/notes to support your scoring. This may aid your reflection at the end of the course

**Importance of Genomic topics to your role**

*5.***Please review each of the topics listed below.**
On a scale of 1 to 5; where '1 is low importance' and '5 is high importance' How important do you feel this knowledge or information is for your role;

Please don't select more than 1 answer(s) per row.

Please select exactly 13 answer(s).

|  | 1 (low importance) | 2 | 3 | 4 | 5 (high importance) | Not applicable |
| --- | --- | --- | --- | --- | --- | --- |
| Understanding of the basic scientific concepts of inheritance, genetics and genomics |  |  |  |  |  |  |
| Understanding of the difference between the germline and somatic (tumour) genome and clinical implications associated with germline or somatic genetic variants |  |  |  |  |  |  |
| Understand what local genetic testing services are available and how to refer patients |  |  |  |  |  |  |
| Ability to carry out appropriate risk assessments to identify patients that might be at a higher risk of inherited conditions |  |  |  |  |  |  |
| Understand the wider roles and services offered by local clinical genetics teams |  |  |  |  |  |  |
| Conduct a comprehensive family history exercise to understand potential high-risk patients for inherited conditions |  |  |  |  |  |  |
| Understand the national genetic test directory and its potential relevance for your patients and practice |  |  |  |  |  |  |
| Understand the targeted therapies available for patients |  |  |  |  |  |  |
| Understand the broad mechanism of action of targeted therapies |  |  |  |  |  |  |
| Understand how genomic data can be used in the context of prevention and earlier diagnosis |  |  |  |  |  |  |
| Understand how genomic data can be used in the context of patient prognosis |  |  |  |  |  |  |
| Understanding how genomic data is analysed and the potential implications of the analysis process on the outcome on patient management |  |  |  |  |  |  |
| Understand the wider legal, social and ethical considerations of genetic testing for patients |  |  |  |  |  |  |

*a.* Please add any comments/notes to support your grading. This may aid your reflection at the end of the course

Page 4: Previous Education and Training in Genetics or Genomics

We would like to know if you have received any education or training in genetics or genomics.

*6.*Please indicate below if you have received education and training by selecting the appropriate boxes below to indicate which, if any, of the following topics you have received education or training on?
Selecting a topic indicates you have received education and training on this topic Please select all that apply. *Required*

Please select between 1 and 14 answers.

 I have not received any education or training on genetics or genomics

 The basic scientific concepts of inheritance, genetics, and genomics

 The difference between the germline and somatic (tumour) genome and clinical implications associated with germline or somatic genetic variants

 How to carry out appropriate risk assessments to identify patients that might be at a higher risk of inherited conditions

 How to conduct a comprehensive family history exercise to understand potential high-risk patients for inherited conditions

 What local genetic testing services are available and how to refer patients

 The wider roles and services offered by local clinical genetics teams

 Information on the national genetic test directory and its potential relevance for your patients and practice

 Information on targeted therapies available for patients within your speciality

 Information on the broad mechanism of action of targeted therapies

 How genomic data can be used in the context of prevention and earlier diagnosis

 How genomic data can be used in the context of patient prognosis

 How genomic data is analysed and the potential implications of the analysis process on the outcome on patient management

 The wider legal, social, and ethical considerations of genetic testing

 Other

*a.* If you selected Other, please specify:

*7.* Finally, please include any comments that you feel are pertinent regarding this topic that you feel you have not had the opportunity to include as part of this survey.

Page 5: You have completed the questionnaire - Thank you!

Thank you for taking time out to complete this survey. The next page will allow you to print a copy of your responses.

Your comments will help our course evaluation and help you in your reflection on your genomics education journey!

We look forward to seeing you here again soon.
